# Ultrathin‐FeOOH‐Coated MnO_2_ Sonosensitizers with Boosted Reactive Oxygen Species Yield and Remodeled Tumor Microenvironment for Efficient Cancer Therapy

**DOI:** 10.1002/advs.202200005

**Published:** 2022-04-28

**Authors:** Qiyu Liu, Liyin Shi, Ying Liao, Xianshuo Cao, Xiaoqing Liu, Yanxia Yu, Zifan Wang, Xihong Lu, Jianwei Wang

**Affiliations:** ^1^ Sun Yat‐Sen University Cancer Center State Key Lab oratory of Oncology in South China Collaborative Innovation Center of Cancer Medicine The Key Lab of Low‐carbon Chem & Energy Conservation of Guangdong Province School of Chemistry Sun Yat‐Sen University Guangzhou 510275 P. R. China; ^2^ Pathology Department of National Cancer Center/National Clinical Research Center for Cancer/Cancer Hospital & Shenzhen Hospital Chinese Academy of Medical Sciences and Peking Union Medical College Shenzhen 518116 P. R. China

**Keywords:** manganese dioxide, reactive oxygen species, sonodynamic therapy, sonosensitizers, tumor microenvironment

## Abstract

Sonodynamic therapy (SDT) typically suffers from compromised anticancer efficacy owing to the low reactive oxygen species (ROS) yield and complicated tumor microenvironment (TME) which can consume ROS and support the occurrence and development of tumors. Herein, ultrathin‐FeOOH‐coated MnO_2_ nanospheres (denoted as MO@FHO) as sonosensitizers which can not only facilitate ultrasound (US)‐triggered ROS but also tune the TME by hypoxia alleviation, H_2_O_2_ consumption as well as glutathione (GSH) depletion are designed. The FeOOH coating will boost the production yield of singlet oxygen (^1^O_2_) and hydroxyl radicals (^•^OH) by inhibiting the recombination of US‐initiated electron–hole pairs and Fenton‐like reaction, respectively. Additionally, the catalase‐like and GSH peroxidase‐like activities of MO@FHO nanospheres enable them to break the TME equilibrium via hypoxia alleviation and GSH depletion. The combination of high ROS yield and fundamental destruction of TME equilibrium results in satisfactory antitumor outcomes, as demonstrated by the high tumor suppression efficacy of MO@FHO on MDA‐MB‐231‐tumor‐bearing mice. No obvious toxicity is detected to normal tissues at therapeutic doses in vivo. The capability to modulate the ROS production and TME simultaneously can afford new probability for the development of advanced sonosensitizers for synergistic comprehensive cancer therapy.

## Graphical abstract

A MnO_2_@FeOOH sonosensitizer is constructed to achieve oncotherapy by modulating reactive oxygen species (ROS) yield and tumor microenvironment. FeOOH coverage acts as hole conductor and O_2_ production cocatalyst to improve ROS production and alleviate hypoxia. The H_2_O_2_ and glutathione depletions are detected by its Fenton‐like and glutathione peroxidase‐like activities. The antitumor efficacy is testified both in vitro and in vivo.

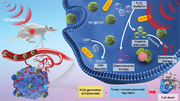

## 1 Introduction

With the ever‐developing nanomedicine, a variety of novel therapeutic methods have been applied to oncotherapy, in addition to traditional surgery, radiotherapy, and chemotherapy. Therein, the demand of good therapeutic effect and few side effects greatly stimulate the boom of minimally invasive or noninvasive therapeutic methods.^[^ ^1^, ^2^, ^3^, ^4^ ^]^ Sonodynamic therapy (SDT), an emerging noninvasive therapeutic modality, is achieved by triggering the sonosensitizers to produce localized cytotoxic reactive oxygen species (ROS) for tumor cell killing.^[^ ^5^, ^6^, ^7^, ^8^ ^]^ This method integrates a series of advantageous properties including minimal damage, deep tissue penetration depth and high spatial precision, and thus attracts increasing research attention. To date, by using SDT, some encouraging achievements have been obtained on various tumor issues like breast and pancreatic tumor at the laboratory level.^[^ ^9^, ^10^, ^11^ ^]^ However, further application of SDT remains severely restricted by its limited anticancer efficacy, even with the most advanced sonosensitizers, including both traditional organic molecules (such as phenothiazine compounds and fluoroquinolone antibiotics) and inorganic nanomaterials (such as noble metal nanoparticles and transition metal oxide).^[^ ^12^, ^13^, ^14^, ^15^, ^16^ ^]^ Therefore, the development of effective sonosensitizers with improved SDT performance is the key for its further clinical transformation.

Currently, increasing the ROS yield of sonosensitizers is the research mainstream to optimize the therapy outcome of SDT. However, it cannot fundamentally solve this problem because the tumor microenvironment (TME), consisting of severe hypoxia, endogenous high levels of H_2_O_2_ (0.1–1 × 10^−3^ m) and overexpressed glutathione (GSH) (1–10 × 10^−3^ m), imposes restrictions on the efficacy of ROS.^[^ ^17^, ^18^, ^19^, ^20^, ^21^ ^]^ To be specific, when SDT is applied to tumor cells, hypoxia is to suppress the production of O_2_‐originated ROS and reductive GSH would also consume additional ROS, both weakening the therapy efficacy.^[^ ^22^, ^23^ ^]^ More importantly, these two factors, combined with highly concentrated H_2_O_2_, create an enabling environment for the genesis, growth and metastasis of tumor cells, even resulting in immune inhibition and multidrug resistance of body to tumor cells.^[^ ^24^, ^25^, ^26^, ^27^ ^]^ Thus, TME regulation has also been adopted recently to optimize the SDT performance.^[^ ^11^, ^28^, ^29^ ^]^ For instance, a hydrogenated hollow Pt‐TiO_2_ Janus was designed to catalyze the decomposition of endogenous H_2_O_2_ to O_2_. The resultant hypoxia alleviation enabled high tumor cell killing efficacy in vitro.^[^ ^30^ ^]^ Furthermore, benefiting from Fenton‐like activity, a TiO_1+_ *_x_* sonosensitizer with rich oxygen‐deficient structure could consume endogenous H_2_O_2_ for hydroxyl radical (^•^OH) production, which greatly improved the therapy outcomes for breast‐tumor‐bearing mice.^[^ ^13^ ^]^ To cope with the superfluous GSH in tumor cells, MnWO_x_ nanoparticles embedding GSH peroxidase‐like activity were developed to convert GSH to glutathione disulfide (GSSG), which effectively impaired the antioxidant capability of tumor cells and thus optimized the SDT efficacy.^[^ ^31^ ^]^ In spite of such inspiring achievements, SDT regulation by tuning TME remains at its infant stage. Especially, most reported work only focused on modulating one aspect of TME, limiting further improvements of the therapy outcome.

**Scheme 1:**
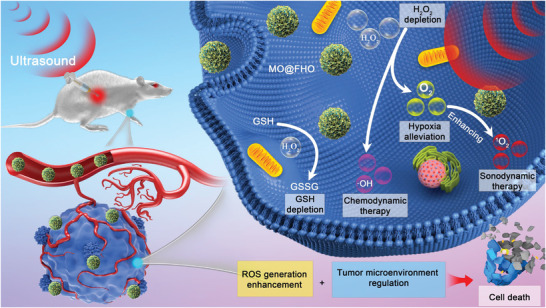
Schematic diagram for the anticancer mechanism of MO@FHO.

Herein, we construct ultrathin‐FeOOH‐coated MnO_2_ nanospheres (denoted as MO@FHO) as bifunctional sonosensitizers which can not only promote the ROS yield but also tune TME from various aspects for highly efficient SDT (**Scheme** **1**). MnO_2_ is designed as the active cores for ultrasound (US)‐initiated ROS generation while the FeOOH coverage, taking account to its specific energy band structure, acts as a hole conductor capable of enhancing the separation of US‐triggered electron–hole pairs to accelerate the ROS production rate. In terms of TME regulation, the tumor hypoxia is effectively relieved by decomposing endogenous H_2_O_2_ into O_2_ benefiting from the catalase‐like property of MnO_2_ core and the O_2_ production cocatalyst function of the FeOOH coverage. In addition, GSH depletion and conversion of H_2_O_2_ to ^•^OH for US‐enhanced chemodynamic therapy (CDT) are achieved simultaneously due to the intrinsic multivalent metal ions (Mn^2+/3+/4+^ and Fe^2+/3+^) of MO@FHO which endows it with GSH peroxidase‐like and Fenton‐like characteristics. As expected, the experimental results well support our predictions. That is, the ROS production yield of MO@FHO is superior to its MnO_2_ counterpart and it can fundamentally disrupt the metabolic equilibrium of tumor cells by interfering with the levels of O_2_, H_2_O_2_ and GSH. The high antitumor efficacy of SDT using MO@FHO is systematically demonstrated both in vitro and in vivo. Moreover, the cytotoxicity of the synthesized MO@FHO nanospheres is negligible at therapeutic doses. The combination of ROS therapy and TME regulation for SDT provides a new clue for future design and development of advanced multifunctional sonosensitizers.

## 2 Results and Discussion

**Figure 1:**
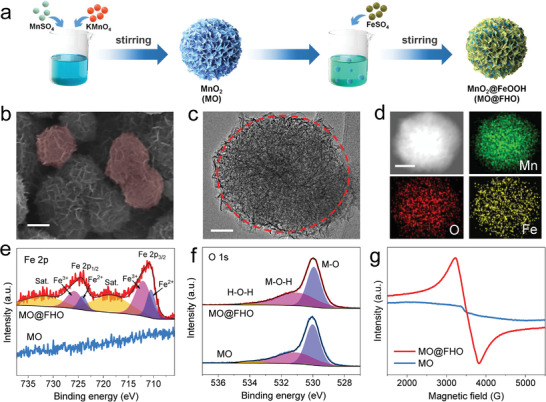
a) Illustration of the synthesis procedure of the MO and MO@FHO. b) SEM image (scale bar: 100 nm), c) TEM image (scale bar: 50 nm), d) TEM (scale bar: 100 nm) and corresponding EDS mapping of the MO@FHO. e) Fe 2p core‐level XPS spectra, f) O 1s core‐level XPS spectra, and g) ESR spectra of the MO and MO@FHO.

MO@FHO nanospheres were synthesized via a two‐step method (**Figure** **1**a). Briefly, MnO_2_ (denoted as MO) was first prepared by a facile liquid precipitation method and an ultrathin FeOOH coverage was then introduced through a replacement reaction.^[^ ^9^, ^32^ ^]^ The morphology and microstructure of MO and MO@FHO were characterized by scanning electron microscopy (SEM) and transmission electron microscopy (TEM). The two materials exhibit a similar nanosphere structure with diameters ranging from 200 to 400 nm, indicating that the presence of FeOOH coverage does not change the morphology of MO (Figure 1b and Figure S1a, Supporting Information). Their size distribution are also illustrated by diameter light scattering (DLS, Figure S2, Supporting Information). Moreover, these nanospheres show a porous structure composed of numerous intersecting nanosheets (Figure 1c and Figure S1b, Supporting Information). TEM and corresponding energy dispersive X‐ray spectroscopy (EDS) elemental mapping reveal that Mn, O, and Fe elements are uniformly distributed in MO@FHO nanospheres (Figure 1d and Figure S1c, Supporting Information). The X‐ray diffraction (XRD) patterns of MO and MO@FHO were measured to examine their crystalline structure (Figure S3, Supporting Information). All the diffraction peaks of MO and MO@FHO agree with Akhtenskite MnO_2_ (*ε*‐MnO_2_, JCPDS No. 30‐0820), and the broad feature corresponds to the characteristic of nanoscale crystalline.^[^ ^33^, ^34^ ^]^ This suggests that there is no phase transition after FeOOH modification, and the mass loading of FeOOH onto MO is quite low and ultrathin. Furthermore, X‐ray photoelectron spectroscopy (XPS) measurements were used to evaluate the chemical composition (Figure S4, Supporting Information) and surface electronic states of the two samples. For MO@FHO, the Fe 2p spectrum (Figure 1e) exhibits two dominant peaks at 710.9 and 724.2 eV accompanied with two satellite (sat.) peaks, corresponding to Fe 2p_3/2_ and Fe 2p_1/2_, respectively. And they can be deconvoluted into two peaks corresponding to Fe^2+^ and Fe^3+^.^[^ ^35^, ^36^ ^]^ The two peaks corresponding to the Mn 2p_3/2_ and Mn 2p_1/2_ peaks (at the binding energy of 641.7 and 653.4 eV, Figure S5a, Supporting Information) are similar for MO and MO@FHO, implying that the FeOOH coverage does not affect the Mn oxidation states.^[^ ^37^ ^]^ In addition, the binding energy difference of 5.0 eV in Mn 3s spectrum indicates the coexistence of Mn^3+^ and Mn^4+^ (Figure S5b, Supporting Information). Moreover, the O 1s spectrum (Figure 1f) is deconvoluted into three peaks at 530.0, 531.1, and 534.1 eV, corresponding to the metal–oxygen (M—O), metal–oxyhydroxide (M—OH) and adsorbed molecular water (H—O—H), respectively.^[^ ^38^, ^39^ ^]^ Apparently, due to the introduction of FeOOH coverage, the amount of M—OH species is increased from 42.0% to 47.3%. The presence of FeOOH on MO@FHO is also confirmed by electron spin resonance (ESR). As shown in Figure 1g, slightly asymmetric signal with Lorentzian shape is observed and its peak‐to‐peak linewidth (Δ*H*_pp_) is about 604 G, which is consistent with FeOOH materials reported in the literature.^[^ ^40^, ^41^ ^]^ As FeOOH coverage is ultrathin for MO@FHO, only the signal of MnO_2_ core is detected by Raman spectra characterization (Figure S6, Supporting Information). Yet, when we extend the reaction time for FeOOH coating to 3 h to increase its thickness (denoted as MO@FHO‐3 h), the typical peaks of FeOOH clearly appear at 209, 274, and 380 cm^−1^.^[^ ^37^, ^42^, ^43^, ^44^ ^]^ Based on the above results, it can be concluded that MO@FHO nanospheres are composed by MnO_2_ core and the ultrathin FeOOH coverage.

**Figure 2:**
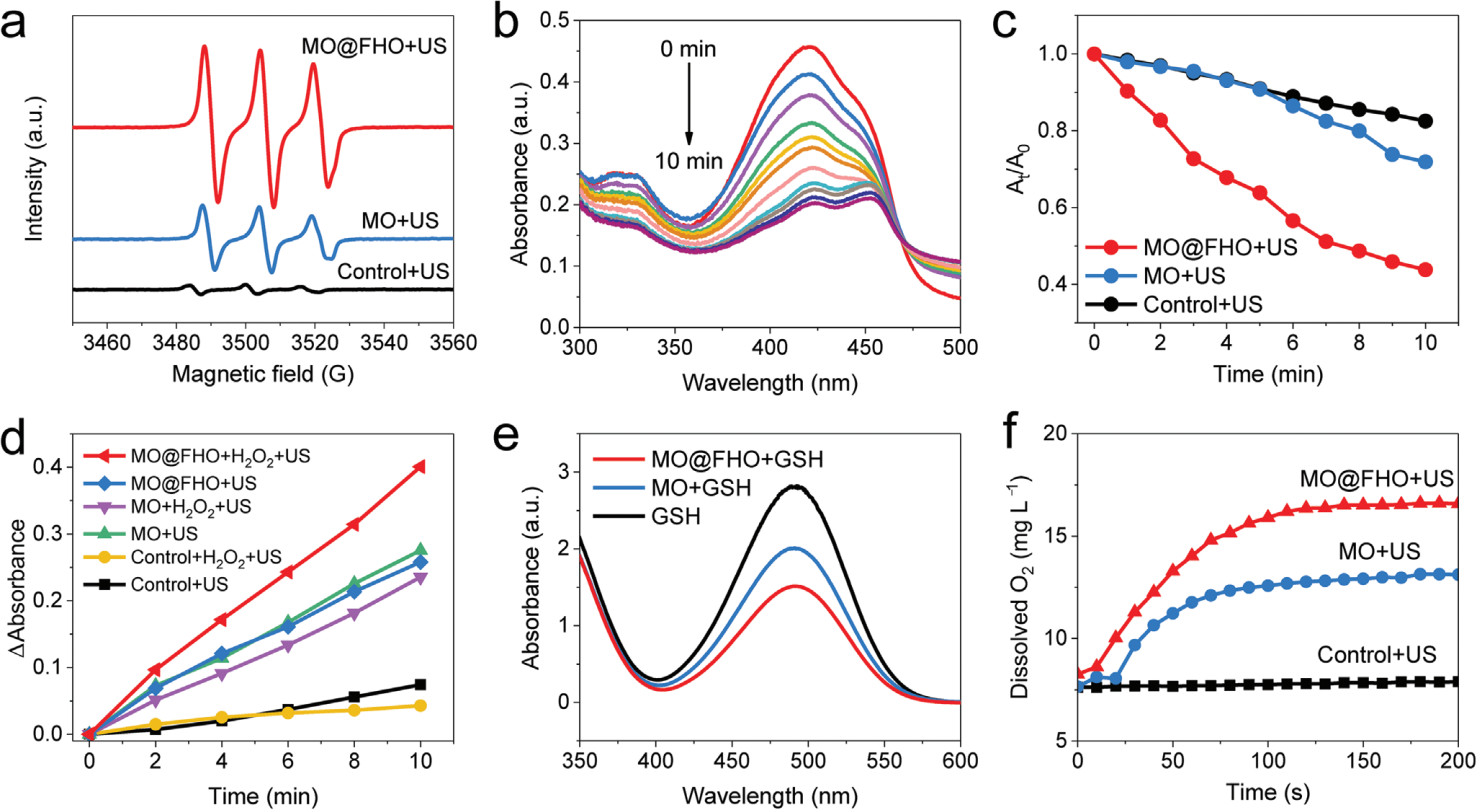
a) ESR spectra of ^1^O_2_ trapped by TEMP of control, MO and MO@FHO under US irradiation for 5 min. b) Time‐dependent UV–vis absorbance spectra of DPBF in the presence of MO@FHO under US irradiation. c) The absorption intensities of DPBF in the presence of MO@FHO as compared with control and MO after different periods of US irradiation. d) The absorption intensities of OPD in different solutions under the US irradiation. e) The absorption intensities of DTNB in different solutions which are incubated in 10^−4^ m GSH for 1 h. f) The detection of O_2_ production of MO and MO@FHO dispersed in PBS solution (0.1 mg mL^−1^, pH = 6.5) with H_2_O_2_ addition (5 × 10^−4^ m).

To compare the ROS yield of MO and MO@FHO, ESR technique was used to examine the generation of singlet oxygen (^1^O_2_) by using 2,2,6,6‐tetramethylpiperide (TEMP) as the trapping agents. As depicted in **Figure** **2**a, under US irradiation, MO@FHO harvests stronger characteristic peak intensity of ^1^O_2_ than control and MO, indicative of its higher ROS yield.^[^ ^45^ ^]^ Similar conclusion was also drawn from UV–vis absorbance measurements with 1,3‐diphenylisobenzofuran (DPBF) as molecular probe (Figure 2b and Figure S7, Supporting Information), where the MO@FHO exhibits the highest DPBF decrease rate under US irradiation among the three samples (Figure 2c).^[^ ^46^ ^]^ In addition to ^1^O_2_, ^•^OH represents another important ROS originating from the oxidation of H_2_O for SDT or from Fenton‐like reaction for CDT. Here, we selected *o*‐phenylenediamine (OPD) as the trapping agent to track the formation of ^•^OH. The similar intensity increment of OPD peak suggests the comparable ability of MO and MO@FHO for oxidizing H_2_O to generate ^•^OH in SDT (Figure S8a–c, Supporting Information).^[^ ^47^ ^]^ ESR results also corroborate the similar ^•^OH yield of MO and MO@FHO with the spin traps of 5,5‐dimethyl‐1‐pyrroline N‐oxide (DMPO) (Figure S9, Supporting Information).^[^ ^48^ ^]^ However, when 10^−4^ m H_2_O_2_ is added to simulate TME, a sharp contrast for ^•^OH generation is observed between the two samples (Figure S8d–f, Supporting Information). The calculated proportion of OPD increase at different irradiation times further confirms that MO@FHO produces a significantly increased amount of ^•^OH with the presence of H_2_O_2_, while MO generates less ^•^OH with or without the presence of H_2_O_2_ (Figure 2d). Such a phenomenon fully proves that MO@FHO is an excellent Fenton‐like reagent to generate ^•^OH for tumor therapy.^[^ ^13^ ^]^

Besides its superior ROS production capability, MO@FHO was also demonstrated to be capable of breaking the TME equilibrium to improve antitumor efficacy, involving hypoxia alleviation, H_2_O_2_ consumption and GSH depletion. First, owing to the presence of the redox couples of Mn (Mn^2+/3+/4+^) and Fe (Fe^2+/3+^), it displays GSH peroxidase‐like activity. When MO@FHO nanospheres are incubated with GSH, the characteristic UV–vis absorbance of 5,5ʹ‐dithiobis (2‐nitrobenzoic acid) (DTNB), an indicator of GSH presence, experiences a sharper decrease than that of MO and GSH alone, demonstrating its higher GSH depletion rate (Figure 2e).^[^ ^20^ ^]^ Second, it can effectively alleviate hypoxia of TME by decomposing intracellular H_2_O_2_ to O_2_, showing catalase‐like activities.^[^ ^49^ ^]^ To verify this point, dissolved O_2_ content increment in real‐time of two samples is detected in PBS solution containing 5 × 10^−4^ m H_2_O_2_ under US irradiation (Figure 2f). After 200 s incubation, the dissolved O_2_ content increment of MO@FHO reaches 8.5 mg L^−1^, about 1.5 times of that of MO (5.7 mg L^−1^). And the calculated ratio of O_2_ production versus the H_2_O_2_ addition of MO and MO@FHO are 0.36 and 0.53, respectively. Furthermore, when the concentration of MO@FHO and H_2_O_2_ increases, the abundant bubbles can be visibly observed, reflecting its high catalase‐like activity more intuitively (Figure S10, Supporting Information). Finally, as previously mentioned, the Fenton‐like activity of MO@FHO enables the consumption of endogenous H_2_O_2_ for ^•^OH production. Namely, instead of tuning one aspect of TME, the as‐designed MO@FHO can simultaneously tuning three factors to break the TME equilibrium and promote the cell apoptosis. More importantly, proper regulation of these three factors is also beneficial for ROS accumulation, thereby synergistically optimizing the therapeutic effect.

**Figure 3:**
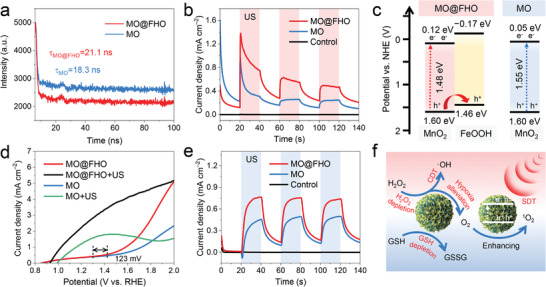
a) Photoluminescence decay profiles of MO and MO@FHO. b) The transient US‐excited current curves in PBS of MO, MO@FHO, and control with a period of 40 s. c) The energy band diagrams and d) LSV curves at 10 mV s^−1^ measured in PBS with or without US irradiation of MO and MO@FHO. e) The transient US‐excited current curves in PBS electrolyte with H_2_O_2_ addition (10^−4^ m) of MO, MO@FHO, and control with a period of 40 s. f) The overall scheme showing mechanisms of MO@FHO sonosensitizer via synergistic effects to achieve the tumor therapy. All US irradiation proceeded under 1 MHz, 1.5 W cm^−2^.

The efficient separation of US‐triggered electron–hole pairs is conducive to producing large amounts of ROS in SDT. To unravel why the ROS yield of MO@FHO is higher than MO, the electron–hole kinetics of the two samples were studied by photoluminescence decay measurements. As illustrated in **Figure** **3**a, the average fluorescence lifetime (*τ*) of MO@FHO (21.1 ns) is longer than that of MO (18.3 ns), indicating that FeOOH coverage can reduce the recombination of electron–hole pairs.^[^ ^50^ ^]^ Similar results were also obtained from amperometric current–time (*I*–*t*) curves at open circuit potential. We can see from Figure 3b that, upon US irradiation, electron–hole pairs are initiated on both MO@FHO and MO, leading to a US‐excited current increase; When the US irradiation is chopped, the recombination of electrons and holes takes place, resulting in a sudden current drop. According to the plots, no response to US irradiation is found from the control sample while prompt and reproducible US‐excited current densities are observed during periodic irradiation for MO@FHO and MO. Yet, the current variation on MO@FHO is more obvious, highlighting the importance of the FeOOH coverage to suppress the electron–hole pairs recombination.

To figure out how FeOOH coverage optimizes the sono‐sensitization effect, we established a model of the energy band structures of the MO and MO@FHO. According to the UV–vis diffuse reflectance spectra and the Kubelka–Munk (KM) formula, the band gaps (*E*_g_) of MO and MO@FHO are similar, being 1.55 and 1.48 eV, respectively (Figure S11, Supporting Information). In addition, the bandgaps for MO@FHO‐3 h (1.63 eV) are also estimated to facilitate model building, which can be considered as pure FeOOH (Figure S12, Supporting Information). Their valence band (VB) potentials are evaluated to be 1.60 eV (MO and MO@FHO) and 1.46 eV (FeOOH) by the VB‐XPS analysis (Figure S13, Supporting Information). And the corresponding conduction band (CB) potentials of MO, MO@FHO and MO@FHO‐3 h are theoretically evaluated from Equation (1)^[^ ^51^, ^52^ ^]^

(1) $$E_{g}=\;\left|E_{\mathrm{CB}}-E_{\mathrm{VB}}\right|$$

Based on the above discussion, the possible energy band diagrams are proposed in Figure 3c. Under US irradiation, electrons and holes are first generated by the MnO_2_ core of MO@FHO. Then, internal electric field drives the holes flow toward FeOOH owing to its more negative conduction band energy level, which significantly promotes the separation of electron–hole pairs.^[^ ^53^ ^]^ That is, FeOOH coverage can function as hole conductor to improve the US‐initiated ROS yield of MO@FHO, thereby optimizing its SDT performance.

Interestingly, we found the FeOOH coverage could also improve the catalase‐like activity of MnO_2_ core, which might benefit the hypoxia alleviation of TME. The linear sweep voltammetry (LSV) curves at 10 mV s^−1^ in PBS solution reveal that, compared to MO, MO@FHO exhibits a much steeper water oxidation current associated with an earlier catalytic onset potential shifted by 123 mV, and the application of US irradiation further enlarges the current response difference (Figure 3d).^[^ ^43^ ^]^ This is because the FeOOH coverage, acting as a cocatalyst, can enhance the reaction kinetics of O_2_ evolution, and the US‐responsive ability of MO@FHO is conducive to the directional effect of the sonosensitizers in vivo.^[^ ^54^ ^]^ In addition, the transient US‐excited current curves in PBS solution containing 10^−4^ m H_2_O_2_ (mimic TME) were measured to distinguish the catalase‐like activity of MO and MO@FHO. In Figure 3e, the MO@FHO electrode presents a higher US‐triggered current pulsation, which is mainly attributed to the cocatalytic effect of the FeOOH layer toward the redox reaction of H_2_O_2_. Namely, due to the presence of FeOOH coverage, more active sites are involved in H_2_O_2_ catalysis and such enhanced catalase‐like activity of MO@FHO is anticipated to effectively alleviate the hypoxia in SDT.

In accordance to our predictions, the smart design of MO@FHO enables it to function as a bifunctional sonosensitizer capable of remarkably boosting the ROS yield and thoroughly breaking TME equilibrium for efficient cancer therapy. The proposed working mechanism of this material is presented in Figure 3f. Briefly, the special energy band structure of the FeOOH coverage greatly raises the ROS yield as it works as a hole conductor to enhance the separation efficiency of US‐excited electron–hole pairs. Moreover, the Fenton‐like activity of the MO@FHO further improves the ROS production for CDT. More importantly, MO@FHO breaks metabolic equilibrium of tumor cells by interfering three factors of TME. Besides the consumption of H_2_O_2_ in CDT, it can also alleviate the hypoxia of TME and deplete GSH taking advantages of its catalase‐like activity and peroxidase‐like characteristic, respectively. These advantageous properties lead to a large accumulation of ROS and synergistical destruction of TME on which cancer cells depend for survival, both of which result in tumor cell death.

**Figure 4:**
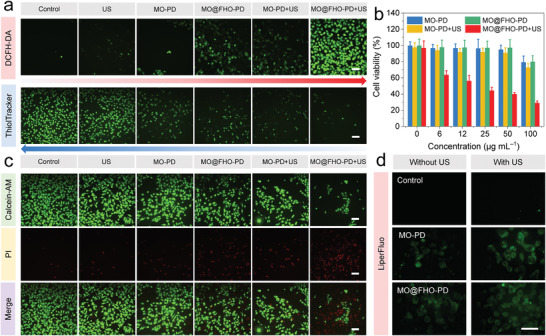
a) CLSM images of MDA‐MB‐231 cells stained with DCFH‐DA to detect ROS level (above) and ThiolTracker Violet to indicate GSH level (below) for differently treated groups. Scale bars: 200 µm. b) Cell viabilities of MDA‐MB‐231 cells with different treatments. c) CLSM images of MDA‐MB‐231 cells co‐stained by Calcein‐AM (live cells) and PI (dead cells). Scale bars: 200 µm. d) CLSM images of MDA‐MB‐231 cells stained with LiperFluo for fluorescent detection of lipid peroxides after differently treatments. Scale bars are 50 µm. All US irradiation proceeded under 1 MHz, 1.5 W cm^−2^, 2 min.

Considering the intricate synergistic effect of MO@FHO under US irradiation, we further assessed its therapeutic effect at the cellular level. Before biological test, the two samples were modified with 1,2‐distearoyl‐sn‐glycero‐3‐phosphoethanolamine‐N‐[methoxy(polyethylene glycol)‐2000] (mPEG‐DSPE) to optimize their dispersion stability and biocompatibility (denoted as MO‐PD and MO@FHO‐PD, respectively). The changes in surface morphology and zeta potential of nanospheres confirm the successful modification of mPGE‐DSPE on the surface of the MO and MO@FHO (Figures S14 and S15, Supporting Information). After modification, the excellent dispersion stability of MO@FHO‐PD is verified by DLS tests (Figure S16, Supporting Information). The cytotoxicity of MO@FHO‐PD was first investigated through the standard Cell Counting Kit‐8 (CCK‐8) assay (Figure S17, Supporting Information). No noticeable cytotoxicity is detected toward normal MCF‐10A cells and MDA‐MB‐231 cancer cells, even at a high concentration (100 µg mL^−1^) after coincubation for 16 h. Afterward, the intracellular ROS levels were directly evaluated by a 2,7‐dichlorodi‐hydrofluorescein diacetate (DCFH‐DA) staining assay under confocal laser scanning microscope (CLSM). Weak intracellular ROS‐related fluorescence is showed in the control group, US‐only group, MO‐PD‐only group, MO@FHO‐PD‐only group, and MO‐PD+US group. In contrast, obvious green fluorescence is observed from cells in the MO@FHO‐PD+US group, suggesting its high intracellular ROS yield (**Figure** **4**a). Afterward, the Thiol Tracker violet staining assay was carried out to assess intracellular GSH levels. Compared to the other groups, the weakest green fluorescence in MO@FHO‐PD+US group is ascribed to the efficient depletion of intracellular GSH.^[^ ^31^ ^]^ The combination of ROS production and TME tuning by GSH depletion enables MO@FHO‐PD to serve as an advanced bifunctional sonosensitizer for highly efficient SDT. Consequently, the in vitro anticancer efficiency of MO@FHO‐PD is satisfactory for MDA‐MB‐231 cells (Figure 4b), significantly surpassing that of MO‐PD. For example, the inhibition rate of cancer cells by MO@FHO‐PD under US irradiation could reach ≈71% at 100 µg mL^−1^, much higher compared to MO‐PD (27%) at the same condition. The superiority of MO@FHO‐PD to MO‐PD for SDT in vitro was also proved by live/dead staining assay (calcein‐AM/PI) (Figure 4c) and flow cytometry apoptosis technique (Figure S18, Supporting Information). Intense increase of lipid peroxides is detected from the dead tumor cells, manifesting that the MO@FHO‐PD might kill the cells by destroying cell membrane integrity and function (Figure 4d and Figure S19, Supporting Information).^[^ ^55^ ^]^ All these results verify that, coupling ROS therapy with TME regulation is an effective way to optimize the SDT efficacy and MO@FHO‐PD is a decent bifunctional sonosensitizer against tumor.

**Figure 5:**
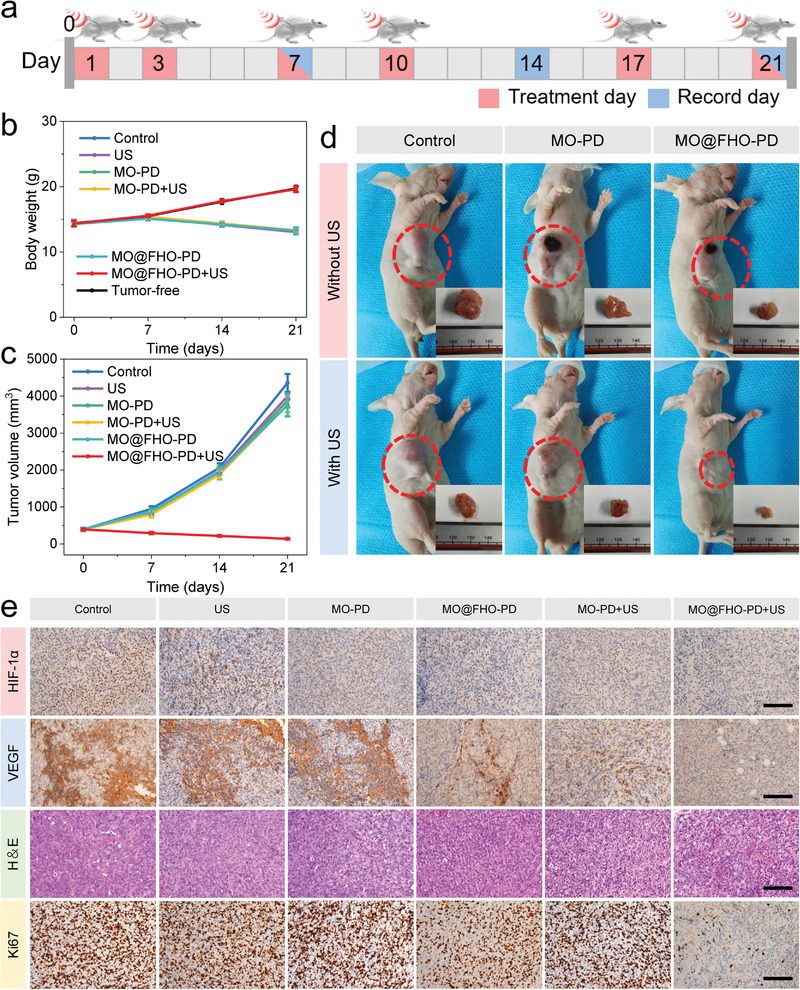
a) Schematic illustration of the procedures against MBA‐MD‐231 tumor on mice. b) Time‐dependent body weight variations of mice after different treatments. c) Time‐dependent tumor volume profile of mice after different treatments. d) Digital images of the nude mice and tumors in the different groups at the end of the treatments. e) HIF‐1*α*, VEGF, H&E, and Ki67 immunohistochemistry staining in tumor region after different treatments. Scale bar: 100 µm.

The excellent antitumor effect of MO@FHO‐PD sonosensitizer motivated us to conduct the in vivo antitumor evaluations toward MBA‐MD‐231 tumor‐bearing female BALB/c nude mice modes. When the subcutaneous tumor diameter reached 3 mm (around 400 mm^3^), the mice were randomly divided to six groups (*n* = 5): 1) control; 2) US only; 3) MO‐PD; 4) MO‐PD+US; 5) MO@FHO‐PD; and 6) MO@FHO‐PD+US. For groups 3 to 6, the dosage of administration was 12.5 mg kg^−1^ while groups 1 and 2 were injected with equal volume of normal saline. After i.v. injection, the tumors of groups 2, 4, and 6 were treated with US irradiation (1 MHz, 1.5 W cm^−2^, 2 min) immediately. And the above treatment was repeated on day 3, 7, 10, 17, and 21 (**Figure** **5**a). After the beginning of treatment, the mice weight and tumor growth of mice during various treatments were recorded every 7 d to evaluate the therapeutic effect. As shown in Figure 5b, the mice in MO@FHO‐PD+US group shows an incremental trend of weight change, which is consistent with the trend of tumor‐free mice, confirming that the adverse effects of the injected dose of MO@FHO‐PD on mice are negligible. In contrast, the mice in other groups undergo a slight weight loss due to the physical discomfort caused by the tumor. As expected, time‐dependent tumor volume profile calculated from the ultrasound images of tumor (Figure S20, Supporting Information) proves that tumor growth is significantly inhibited in mice treated with MO@FHO‐PD+US, demonstrating the satisfactory therapeutic effect of MO@FHO‐PD (Figure 5c). The digital images of the tumor‐bearing mice and tumors also testified such impressive results (Figure 5d).

To verify whether MO@FHO‐PD can alleviate the tumor hypoxia in vivo by virtue of its excellent catalase‐like activity, at the end of the treatments on the mice, we detected hypoxia‐inducible factor (HIF‐1*α*) and vascular endothelial growth factor (VEGF), two indicators closely related to tumor hypoxia, by immunostaining assays.^[^ ^30^, ^56^ ^]^ Obviously, the hypoxia signal (brown part in Figure 5e) of tumor slices in the MO@FHO‐PD+US group is weaker than that of the other groups, especially compared to the control group, suggesting its superior ability for tumor hypoxia alleviation. Such advantageous characteristic can decrease hypoxia‐associated SDT resistance and alter the tumor‐dependent environment, resulting in better therapy outcome. Hematoxylin‐eosin (H&E) and Ki67 observation of tumor section confirms severe apoptosis and inhibition of cell proliferation in MO@FHO‐PD+US group.^[^ ^57^, ^58^ ^]^ H&E immunochemical staining assay was performed on the main organs of mice in each group after 21 d, including heart, liver, spleen, lung, and kidney (Figure S21, Supporting Information). No evident damage or inflammatory response is observed after various treatments, which further proves the excellent biosafety of MO@FHO‐PD in vivo.

## 3 Conclusion

In summary, MO@FHO nanospheres are constructed as advanced bifunctional sonosensitizers which achieve effective antitumor efficacy by simultaneously raising ROS yield and tuning TME. For ROS enhancement, the FeOOH coverage promotes the ^1^O_2_ production by inhibiting the recombination of US‐triggered electron–hole pairs as a hole conductor and the Fenton‐like activity of Fe species facilitates the generation of ^•^OH derived from endogenous H_2_O_2_. For TME regulation, MO@FHO sonosensitizers are capable of alleviating the tumor hypoxia, consuming endogenous H_2_O_2_ and depleting GSH, interfering with the favorable environment for the genesis, growth and metastasis of tumor cells. Further experiments in vivo and in vitro unravel that, taking advantages of the raised ROS yield and TME regulation, the MO@FHO‐PD modified by mPEG‐DSPE elicits robust antitumor effect. The sonodynamic killing efficacy of MDA‐MB‐231 cells reaches ≈71% at 100 µg mL^−1^ and the tumor growth of MDA‐MB‐231‐tumor‐bearing mice is effectively inhibited after 21‐d treatment, posing no obvious side effects. This work highlights a new strategy to combine ROS therapy and TME adjustment through a series of synergistic effects, bringing new opportunities in the development of multifunctional sonosensitizers.

## Conflict of Interest

The authors declare no conflict of interest.

## Supporting information

Supporting Information

## Data Availability

The data that support the findings of this study are available from the corresponding author upon reasonable request.
