# Supplementary material for: Ultrathin‐FeOOH‐Coated MnO2 Sonosensitizers with Boosted Reactive Oxygen Species Yield and Remodeled Tumor Microenvironment for Efficient Cancer Therapy
Source: Adv Sci (Weinh). 2022 Apr 28;9(17):2200005. doi: 10.1002/advs.202200005 (PMC9189684; doi:10.1002/advs.202200005)
Supplement: Supplementary file 1 — Supporting Information [file ADVS-9-2200005-s001.pdf]

ADVANCED SCIENCE
Open Access

Supporting Information

for *Adv. Sci.*, DOI 10.1002/advs.202200005

Ultrathin-FeOOH-Coated MnO_2 Sonosensitizers with Boosted Reactive Oxygen Species Yield and Remodeled Tumor Microenvironment for Efficient Cancer Therapy

Qiyu Liu, Liyin Shi, Ying Liao, Xianshuo Cao, Xiaoqing Liu, Yanxia Yu, Zifan Wang, Xihong Lu and Jianwei Wang**

ADVANCED SCIENCE
Open Access

Supporting Information

for *Adv. Sci.*, DOI: 10.1002/advs202200005

Ultrathin-FeOOH-Coated MnO_2 Sonosensitizers with Boosted Reactive Oxygen Species Yield and Remodeled Tumor Microenvironment for Efficient Cancer Therapy

Qiyu Liu, Liyin Shi, Ying Liao, Xianshuo Cao, Xiaoqing Liu, Yanxia Yu, Zifan Wang, Xihong Lu and Jianwei Wang**

Supporting Information

Ultrathin-FeOOH-Coated MnO_2 Sonosensitizers with Boosted Reactive Oxygen Species Yield and Remodeled Tumor Microenvironment for Efficient Cancer Therapy

Qiyu Liu, Liyin Shi, Ying Liao, Xianshuo Cao, Xiaoqing Liu, Yanxia Yu, Zifan Wang, Xihong Lu and Jianwei Wang**

Experiment Section

Preparation of MO and MO@FHO： All the chemicals were used directly without further purification. The MO@FHO was prepared by a two-step method. The first step was based on a comproportionation reaction. First, 15 mmol of $MnSO_4 \cdot H_2O$ was dispersed in 450 mL of deionized water under magnetic stirring to obtain a transparent pink solution, and then 37.5 mmol of $KMnO_4$ was added slowly. After stirring for 3 h, the MnO_2 powder was obtained through vacuum filtration and repeatedly washed with deionized water. Finally, the produced power was dried overnight at 60 °C (denoted as MO). On this basis, the replacement reaction was used to prepare MO@FHO. 100 mg of MO powder was added to 20 mL of freshly $FeSO_4$ aqueous solution (20 mM) for 100 s, then the resulting powder was centrifuged, washed with deionized water and dried overnight to derive MO@FHO.

Synthesis of MO-PD and MO@FHO-PD： MO or MO@FHO was ground with 1,2-distearoyl-sn-glycero-3-phosphoethanolamine-N-[methoxy(polyethylene glycol)-2000] (mPEG-DSPE) to mix, respectively (mass ratio: $M_{MO\ or\ MO@FHO}$:$M_{mPEG\text{-}DSPE}$=1:5). After that, mixed materials were dispersed in Dulbecco's modified eagle medium (DMEM) basic by ultrasonication (US) for 30 min and then stored in 4 °C for further use.

Fabrication of control, MO and MO@FHO electrodes: To carry out the electrochemical measurements, two samples should be need to be prepared as electrodes. To be specific, MO and MO@FHO powders were mixed with polyvinylidene fluoride binder (PVDF, AR, Sigma-Aldrich), respectively. The weight ratio was 9:1 and 1-methyl-2-pyrrolidinone (NMP, AR, Sigma-Aldrich) was used as the solvent. The mixture was painted on a carbon paper (Shanghai Hesen Electric Company). After drying overnight in vacuum at 60 °C, the electrodes were obtained. And the control sample without active material (MO and

MO@FHO powders) was prepared as the control electrode. The total mass loadings of three electrodes were all about 2.3 mg cm^{-2}.

Characterization: Field-emission scanning electron microscopy (SEM, JSM-6330F and g-500) and transmission electron microscopy (TEM, FEI Tecnai G^2 F30) were used to character the morphology and microstructure of MO and MO@FHO. Element mapping (FEI Tecnai G^2 F30), X-ray diffraction (XRD, D-MAX 2200 VPC, RIGAKU), X-ray photoelectron spectroscopy (XPS, NEXSA, Thermo VG), electron spin resonance spectrometer (ESR, A300, Bruker) and Raman spectroscopy (Renishaw inVia) were used to character the element, composition and valence of MO and MO@FHO. UV-vis absorption spectra were quantified by UV-vis-NIR spectrophotometer (Lambda 950, Perkin Elmer). The US generator was made by DCT-700, SZWELLD. Photoluminescence decay profiles was detected by FLS980, EDINBURGH INSTRUMENTS. Zeta potential was determined on Brookhaven Zetasizer Nanoseries (EliteSizer) using deionized water as medium. Particle sizes of MO-PD and MO@FHO-PD were measured on dynamic light scattering (DLS, NanoBrook 90Plus PALS) using undiluted Research Grade EU Approved Serum as medium.

ROS generation under ultrasound irradiation: Objective to $^1O_2$ detection, 3 mL of MO@FHO solution (0.05 mg mL^{-1} in deionized water) was mixed with 10 μL of 1,3-diphenylisobenzofuran (DPBF, 10 mM in DMSO). Then the mixture was exposed to US irradiation at fixed time intervals (1 min) until 10 min. The concentration of DPBF at different time points was recorded by UV-vis spectra. For •OH detection, 3 mL of MO@FHO solution (0.05 mg mL^{-1}) was mixed with 10 μL of o-phenylenediamine (OPD, 0.1 M in deionized water). Then the mixture was exposed to US irradiation at fixed time intervals (2 min) until 10 min. UV-vis spectra were measured to monitor the ascending trend at different time points. And ESR technology was also employed to detect the generated ROS. In this case, 100 μL of MO@FHO solution (0.1 mg mL^{-1}) was mixed with 20 μL of 2,2,6,6-tetramethylpiperidine (TEMP, for $^1O_2$ detection) or 30 μL of 5,5-dimethyl-pyrroline-N-oxide (DMPO, for ·OH detection). The mixture was exposed to US irradiation for 5 min and immediately detect characteristic peak signals by ESR technology. In addition, the reactive oxygen species (ROS) generating ability of control (without sonosensitizers) and MO groups was detected by the same way. All experiments were carried out under fixed US parameters (1 MHz, 1.5 W cm^{-2}).

Fenton-like activity: 3 mL of MO@FHO solution (0.05 mg mL^{-1} in deionized water) was incubated with H_2O_2 (10^{-4} M), and 10 μL OPD (0.1 M in deionized water) was added as an indicator of •OH. Then the mixture was exposed to US irradiation (1 MHz, 1.5 W cm^{-2}) at fixed time intervals (2 min) until 10 min. UV-vis spectra of the samples at different time

points were scanned to monitor the ascending trend. The Fenton-like activity measurement of control and MO groups was detected by the same way.

GSH peroxidase-like activity: 3 mL of MO@FHO solution (0.05 mg mL^{-1} in dimethyl sulfoxide) was incubated with glutathione (GSH, 10^{-4} M) for 1 h, and then 100 μL 5,5′-dithiobis (2-nitrobenzoic acid) (DTNB, 3 mg mL^{-1} in dimethyl sulfoxide) was added as an indicator of GSH. After standing for 1min, UV-vis spectra of the sample were scanned to monitor the GSH depletion. The GSH peroxidase-like activity measurement of control and MO groups was detected by the same way.

Catalase-like activity: The H_2O_2 (5×10^{-4} M) was added into the phosphate buffered saline (PBS, pH=6.5) solution of MO@FHO (0.1 mg mL^{-1}). Then, dissolved O_2 content increment in real-time was monitored by a portable dissolved oxygen meter (JPSJ-605F, INESA). The catalase-like activity measurement of control and MO groups was detected by the same way.

Electrochemical measurements: Amperometric current-time (I-t) and linear sweep voltammetry (LSV) curves were collected by using an electrochemical workstation (CHI 760). All the electrochemical measurements were characterized in a three-electrode system, in which PBS with/without H_2O_2 addition (10^{-4} M) as the electrolyte, saturated calomel as the reference electrode and graphite rod as the counter electrode.

In vitro cytotoxicity assay: Normal MCF-10A cells or MDA-MB-231 cancer cells were seeded into each well in a 96-well black plate at the density of 1×10^4 $well^{-1}$ and then incubated for 12 h for attachment at 37 °C under 5% CO_2. Subsequently, cells were incubated with different concentration of MO@FHO-PD (0-100 μg mL^{-1}) for another 16 h. Finally, the relative cell viabilities were determined by CCK-8 assay. The relative cell viability was calculated by the equation:

$$\text{Cell viability (\%)} = \frac{OD_{\text{treatment group}} - OD_{\text{Blank group}}}{OD_{\text{control group}} - OD_{\text{Blank group}}} \times 100 \quad (1)$$

In vitro ROS generation and GSH depletion: MDA-MB-231 cancer cells were seeded into each well in a 96-well black plate at the density of 1×10^4 $well^{-1}$ and then incubated for 12 h for attachment at 37 °C under 5% CO_2. Subsequently, cells were co-incubated with different treatments including control, US (1 MHz, 1.5 W cm^{-2}, 2 min), MO-PD (10 μg mL^{-1}), MO@FHO-PD (10 μg mL^{-1}), MO-PD+US and MO@FHO-PD+US for another 16 h. Intracellular ROS levels were detected by the ROS assay kit and intracellular GSH content were assessed by the ThiolTracker Violet. Finally, the intracellular florescence signals were detected by fluorescence Microscope (OLYMPUS IX71).

In vitro antitumor efficacy: MDA-MB-231 cancer cells were seeded into each well in a 96-well black plate at the density of 1×10^4 well^{-1} and then incubated for attachment at 37 °C under 5% CO_2. Subsequently, cells were co-incubated with different concentration of MO@FHO-PD (0-100 μg mL^{-1}) for another 16 h. Every 8 hours, the cells were exposed to a low-intensity ultrasound at 1 MHz for 2 min. Finally, the relative cell viabilities were determined by CCK-8 assay. The relative cell viability was calculated by the equation (1). Similar, the relative cell viabilities of other different treatments were tested by the same method including MO-PD, MO-PD+US and MO@FHO.

Live-dead cell staining assay: The live-dead cell staining assays were carried out to verify the cytotoxicity of MO-PD and MO@FHO-PD. The cell inoculation and treatment processes were the same as above in vitro ROS generation and GSH depletion mentioned. After different treatments, the PI (4.5 μM) and medium containing calcein-AM (2 μM) were added. After another 15 min of culture, the cells were imaged by fluorescence Microscope (OLYMPUS IX71).

Cell apoptosis analysis: Flow cytometry was used to examine cell apoptosis analysis. MDA-MB-231 cancer cells were seeded on 6-well culture plates at an initial density of 1×10^5 cells/well and incubated overnight. cells were co-incubated with different treatments including control, US (1 MHz, 1.5 W cm^{-2}, 2 min), MO-PD (10 μg mL^{-1}), MO@FHO-PD (10 μg mL^{-1}), MO-PD+US and MO@FHO-PD+US for another 16 h. Finally, the cell apoptosis of different treatments was detected by Annexin V, FITC kit.

Lipid peroxides analysis: The cell inoculation and treatment processes were the same as above in vitro ROS generation and GSH depletion mentioned. After different treatments. Subsequently, Liperfluo (15 μM) were added to the wells for 60 min. Then remove the solution and wash twice with Hank's balanced salt solution (HBSS). Finally, the cells were imaged by fluorescence Microscope (OLYMPUS IX71).

Animal Experiments: Healthy female Balb/c nude mice (4-5 weeks old, 14-15 g) were purchased from Guangdong Animal Experiment Center. The use of animals has been approved by the Institutional Animal Care and Use Committee of Sun Yat-Sen University Cancer Center (approval number: L102012019220G). Then, 0.2 mL of MBA-MD-231 cells suspension (2×10^7 mL^{-1}) was injected subcutaneously into the right axilla of each nude mice. When the subcutaneous tumor diameter reached 3 mm (around 400 mm^3) after 14 days, the MBA-MD-231 tumor-bearing mice were randomly divided to six groups (n=5) and start the experiment: (1) control; (2) US only; (3) MO-PD; (4) MO-PD+US; (5) MO@FHO-PD; and (6) MO@FHO-PD+US.

On day 0 of the experiment, the nude mice were anesthetized by inhaling 2% animal isoflurane. After inhalation anesthesia, ultrasound images were used to calculate the tumor volumes of each nude mice by the following equation:

$$\text{Volume} = (\text{transverse diameter})^2 \times \text{vertical diameters} \times 3\pi/4 \quad (2)$$

Then groups 3 and 4 were injected with 12.5 mg kg^{-1} of MO-PD, groups 4 and 6 were injected with 12.5 mg kg^{-1} of MO@FHO-PD, and groups 1 and 2 were injected with equal volume of normal saline. After i.v. injection, the tumors of groups 2, 4 and 6 were treated with US irradiation (1 MHz, 1.5 W cm^{-2}, 2 min) immediately. The procedures were repeated on day 3, 7, 10, 17, and 21 respectively, and the tumor volumes on day 0, 7, 14, and 21 were recorded.

On day 21 of the experiment, all nude mice were euthanized with excessive anesthesia and dissected. The heart, lung, liver, spleen and kidney of each group were collected for Hematoxylin-eosin (H&E) observation. And tumor tissues were collected to execute H&E, vascular endothelial growth factor (VEGF), Ki67 and hypoxia-inducible factor (HIF-1α) immunohistochemistry staining.

Statistical Analysis: All experiments were performed at least three times, while the results are presented as mean ± SD. The significant difference between different groups was calculated by Two-way ANOVA with Bonferroni posttests. Significance is indicated by $P < 0.05$ (*$P < 0.05$, **$P < 0.01$, ***$P < 0.001$). All data were analyzed by GraphPad Prism and Excel.

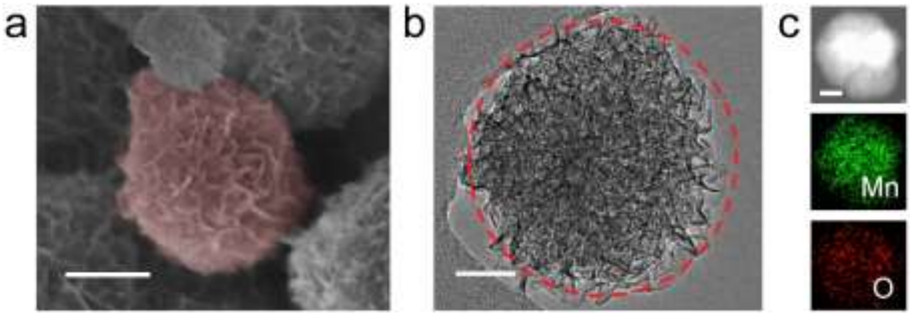

Figure S1. a) SEM image (scale bar: 100 nm), b) TEM image (scale bar: 50 nm), c) TEM (scale bar: 100 nm) and corresponding EDS mapping of the MO.

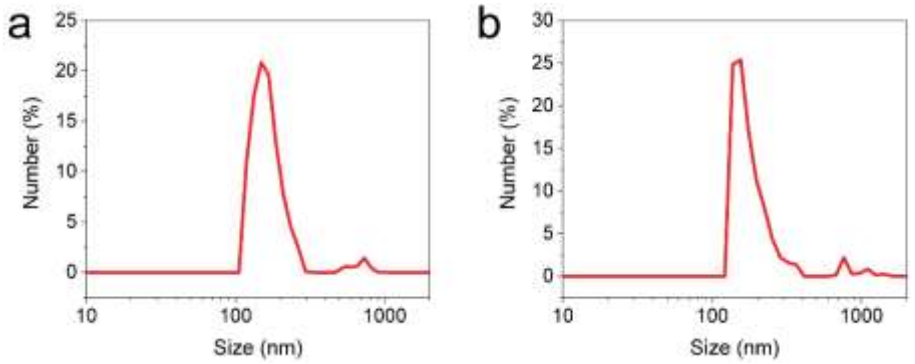

Figure S2. Size distributions of a) MO and b) MO@FHO measured by DLS.

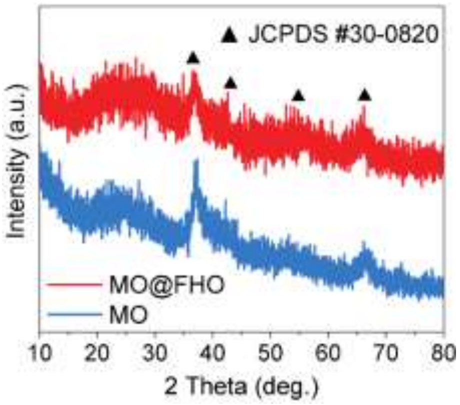

Figure S3. XRD patterns of MO and MO@FHO.

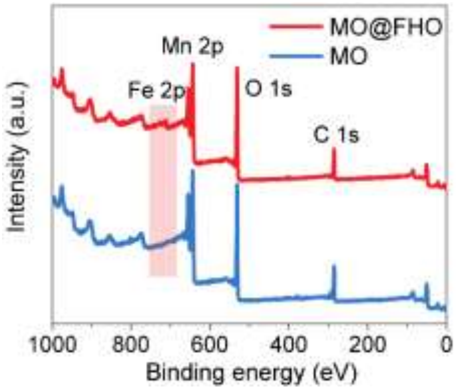

Figure S4. XPS survey spectra of MO and MO@FHO. The existence of C element is due to the adsorption of adventitious organic small molecules in the air.

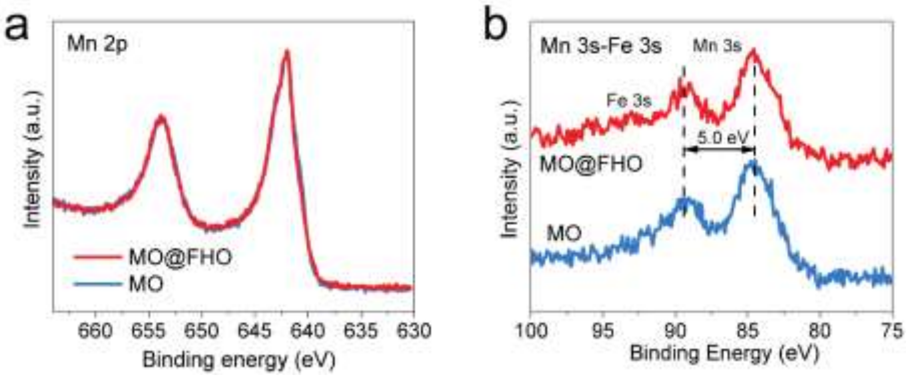

Figure S5. a) Mn 2p and b) Mn 3s-Fe 3s core-level XPS spectra of MO and MO@FHO.

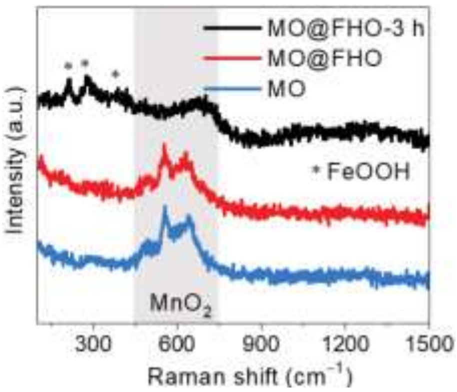

Figure S6. Raman spectra of MO, MO@FHO and MO@FHO-3 h.

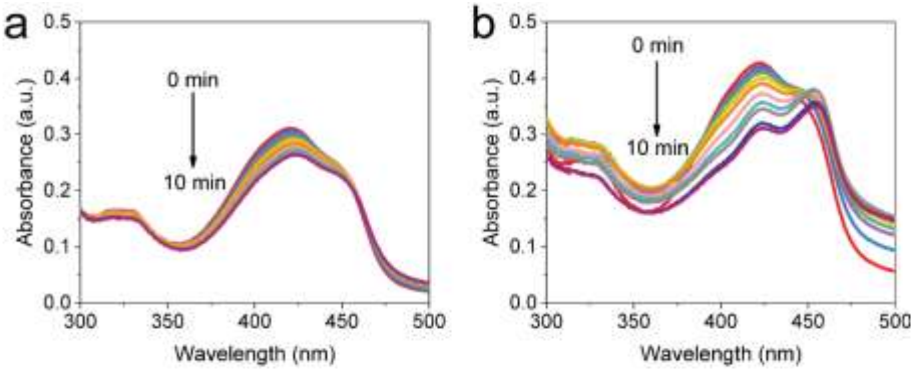

Figure S7. Time-dependent UV-vis absorbance spectra of DPBF a) without sonosensitizer (control) and b) in the presence of MO under US irradiation.

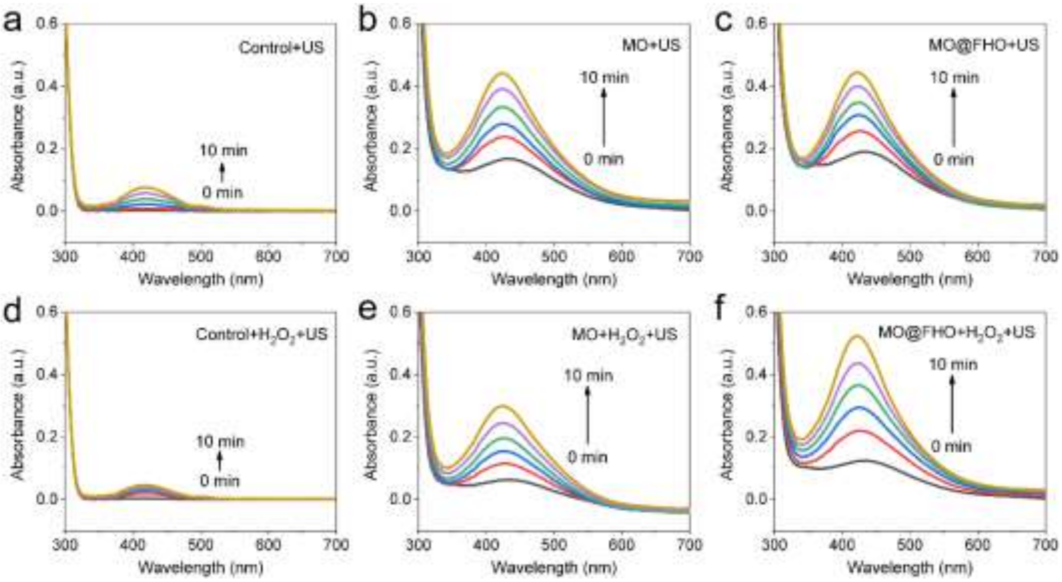

Figure S8. Time-dependent UV-vis absorbance spectra of OPD a) without sonosensitizer (control) and in the presence of b) MO and c) MO@FHO under US irradiation. Time-dependent UV-vis absorbance spectra with additional H_2O_2 (10^{-4} M) of d) control, e) MO and f) MO@FHO under US irradiation with OPD as the trapping agent.

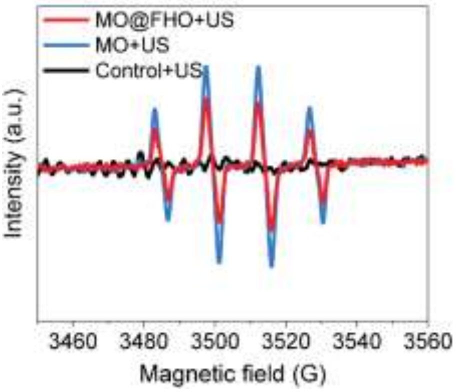

Figure S9. ESR spectra of ·OH trapped by DMPO of control, MO and MO@FHO under US irradiation for 5 min.

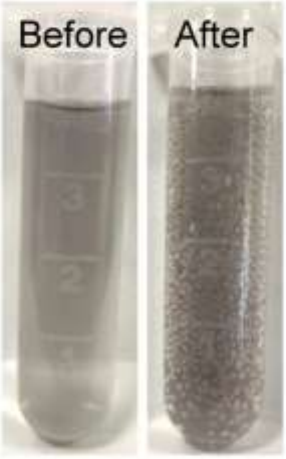

Figure S10. O_2 generation photographs of MO@FHO before and after adding H_2O_2. The 4 mL of MO@FHO solution (0.05 mg mL^{-1} in deionized water) is placed in a centrifuge tube, and subsequently adding 40 μL of H_2O_2 solution (1 M in deionized water).

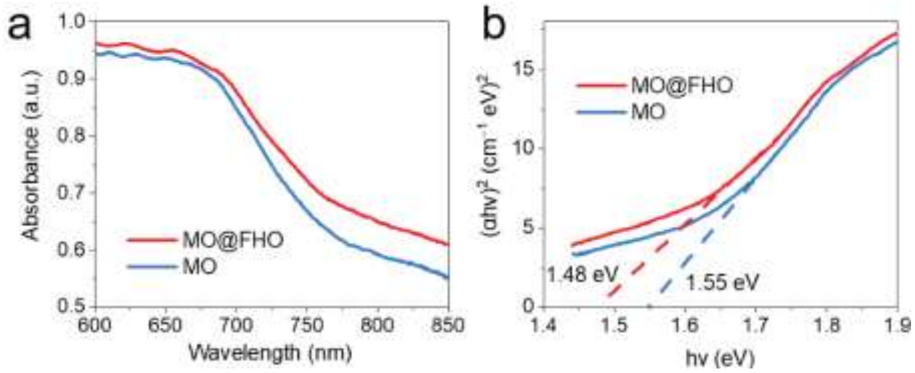

Figure S11. a) UV-vis diffuse reflectance spectra and b) calculated band gap patterns based on UV-vis diffuse reflectance spectra of MO and MO@FHO.

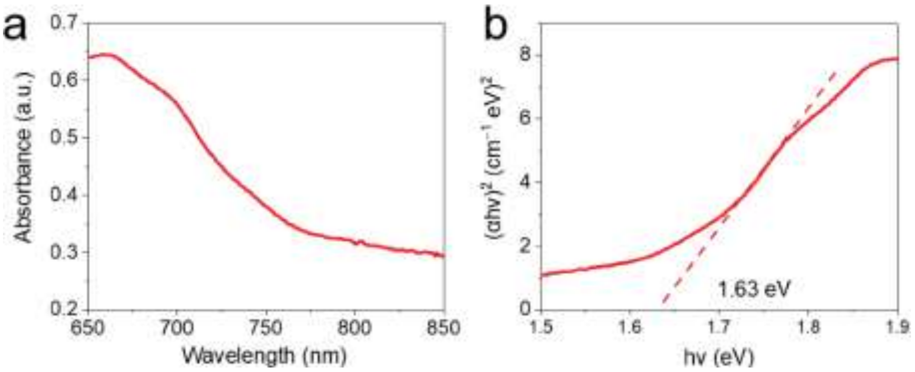

Figure S12. a) UV-vis diffuse reflectance spectra and b) calculated band gap patterns based on UV-vis diffuse reflectance spectra of MO@FHO-3h.

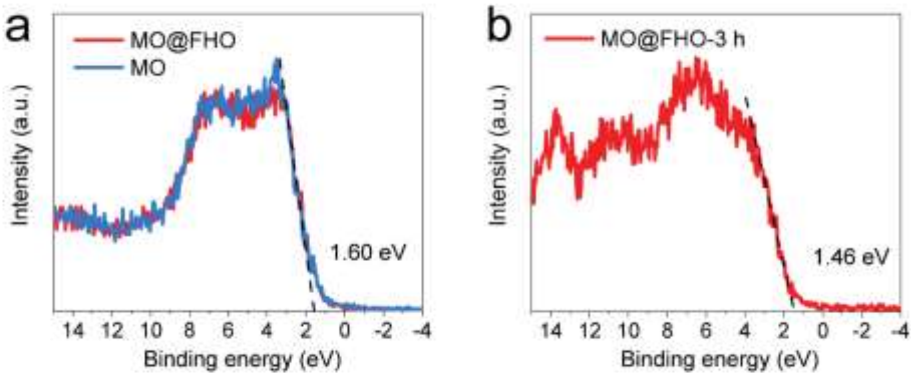

Figure S13. XPS valence band spectrum of a) MO, MO@FHO and b) MO@FHO-3 h.

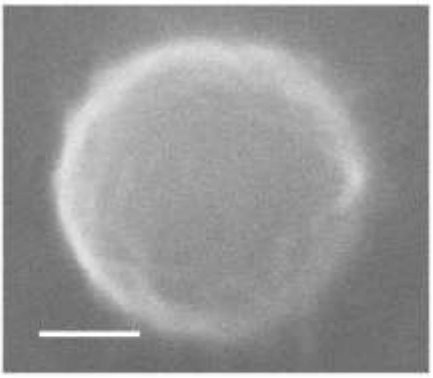

Figure S14. SEM image of MO@FHO-PD (scale bar: 100 nm). The surface of MO@FHO-PD becomes smooth instead of porous.

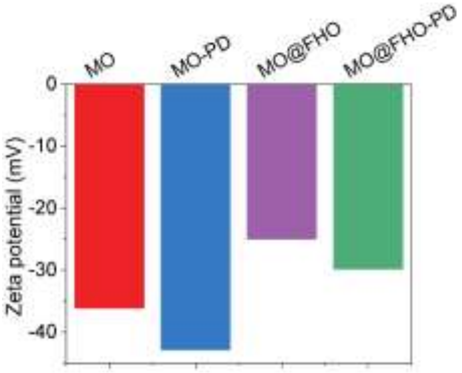

Figure S15. Zeta potential of MO, MO@FHO, MO-PD and MO@FHO-PD. The zeta potential of MO@FHO changes from −25.0 to −29.8 mV, while the zeta potential of MO decreases from −36.1 to −42.9 mV after the modification of PGE-DSPE.

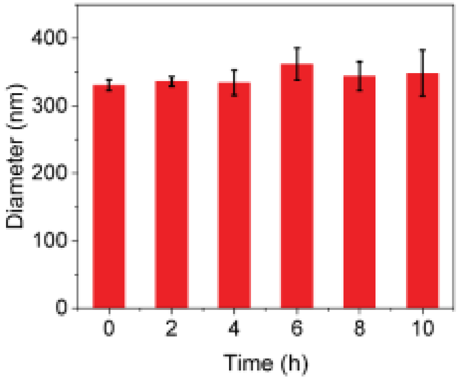

Figure S16. Size distribution at different standing times (0-10 h) of MO@FHO-PD dispersed in Research Grade EU Approved Serum.

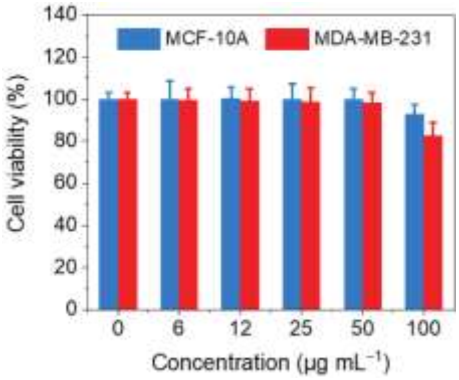

Figure S17. Relative viabilities of normal MCF-10A cells and MDA-MB-231 cancer cells after incubation with varied concentrations of MO@FHO-PD for 16 h.

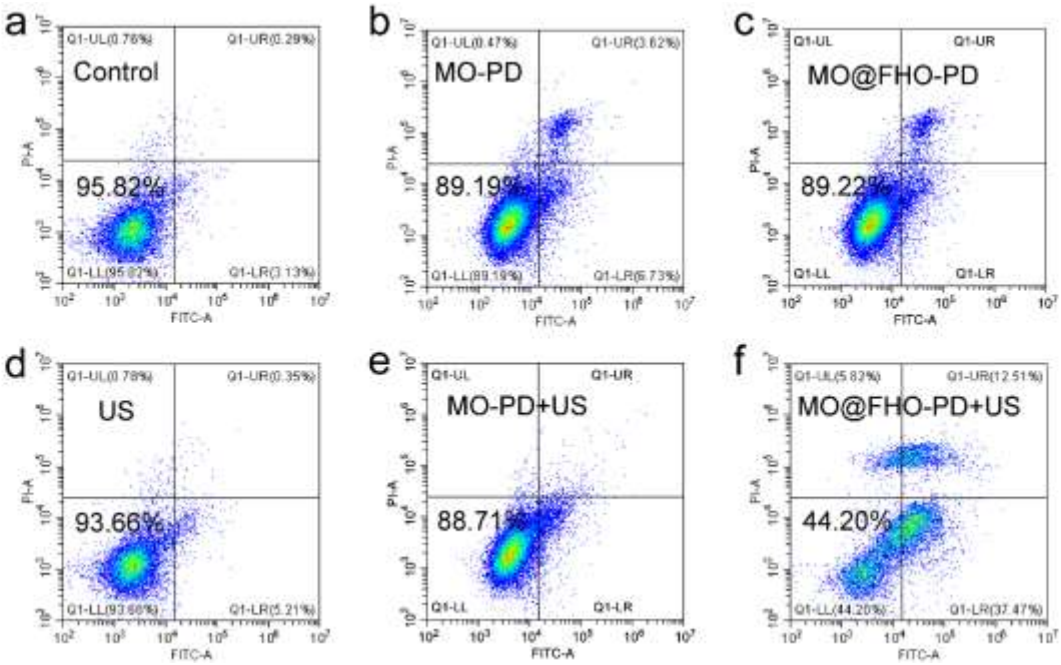

Figure S18. Flow cytometry analysis of MDA-MB-231 cells apoptosis subjected to different treatments.

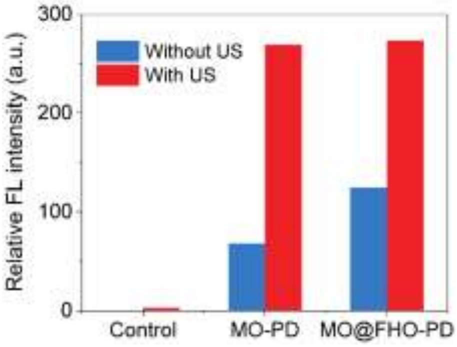

Figure S19. Mean fluorescence intensity determined by ImageJ software of lipid peroxides variation after differently treatments.

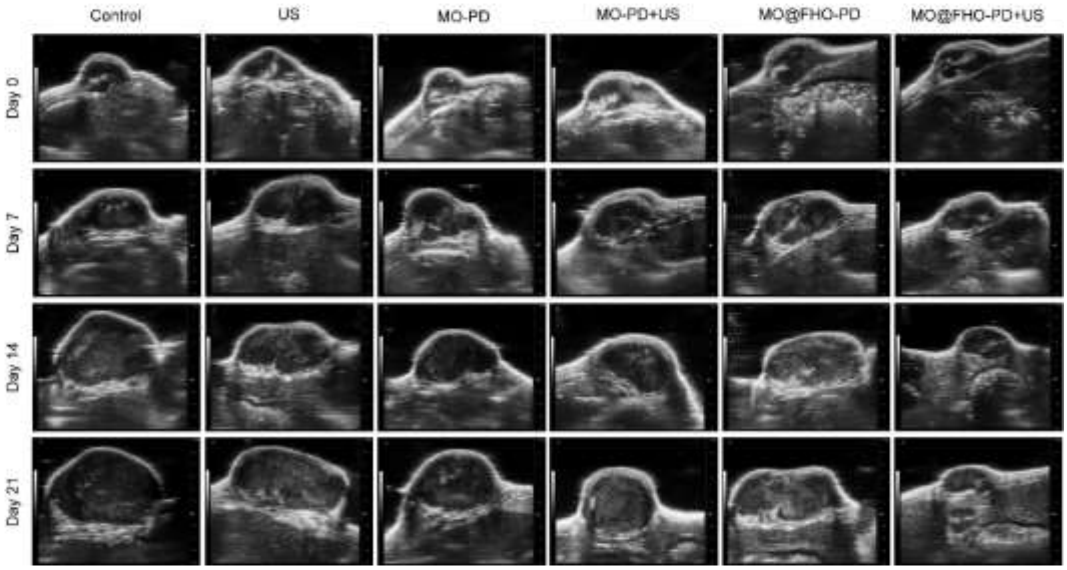

Figure S20. Ultrasound images of MBA-MD-231 tumor in the different groups at different days.

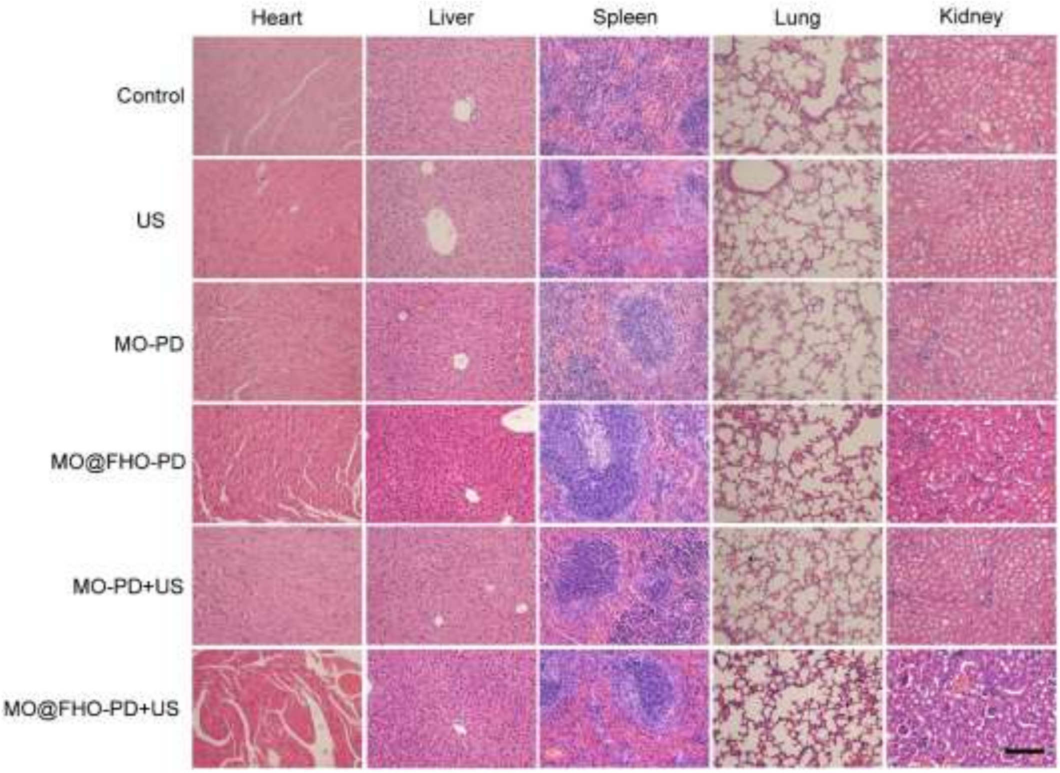

Figure S21. H&E staining of main organs (heart, liver, spleen, lung and kidney) in different groups after the end of treatment. Scale bar: 100 μm.
